# Supplementary material for: Persistent humoral and CD4+ TH cell immunity after mild SARS-COV-2 infection—The CoNAN long-term study
Source: Front Immunol. 2023 Jan 13;13:1095129. doi: 10.3389/fimmu.2022.1095129 (PMC9880277; doi:10.3389/fimmu.2022.1095129)
Supplement: Supplementary file 1 [file DataSheet_1.pdf]

PERSISTENT IMMUNITY AFTER MILD SARS CoV-2 INFECTION

# - THE CoNAN-LONG TERM STUDY –

SUPPLEMENTAL MATERIAL

Clara Schnizer[1*], Nico Andreas[2*], Wolfgang Vivas[1,3], Thomas Kamradt[2], Michael Baier[4], Michael Kiehntopf[5], Stefan Glöckner[4], André Scherag[6], Bettina Löffler[4], Steffi Kolanos[1], Joel Guerra[1,7] Mathias W. Pletz[1,8#], Sebastian Weis[1,3,7#] and the CoNAN study group.

[*#] contributed equally

[1] Institute for Infectious Diseases and Infection Control, Jena University Hospital- Friedrich Schiller University, Jena, Germany

[2] Institute of Immunology, Jena University Hospital- Friedrich Schiller University, Jena, Germany

[3] Leibniz Institute for Natural Product Research and Infection Biology – Hans Knöll Institute (HKI), Jena, Germany

[4] Institute of Medical Microbiology, Jena University Hospital- Friedrich Schiller University, Jena, Germany

[5] Institute of Clinical Chemistry and Laboratory Diagnostics and Integrated Biobank Jena (IBBJ), Jena University Hospital- Friedrich Schiller University, Jena, Germany

[6] Institute of Medical Statistics, Computer and Data Sciences, Jena University Hospital- Friedrich Schiller University, Jena, Germany

[7] Department of Anesthesiology and Intensive Care, Jena University Hospital- Friedrich Schiller University, Jena, Germany

[8] Center for Sepsis Control and Care, Jena University Hospital- Friedrich Schiller University, Jena, Germany

SUPPLEMENTAL MATERIAL

Supplementary Figure 1 (refers to *Figure 2*): **A**) Ratios of anti-SARS-CoV-2 antibody levels over time from the indicated tests. **B,C**) ofaAnti-SARS-CoV-2 antibody levels over time as assessed with **B**) the quantitative Diasorin and **C**) the Euroimmun test (OD-ratio) and stratified by age, symptomatic disease, PCR status during the outbreak and sex. Dots represent individual participants. N= number of individuals per group. Friedman test with Dunns post-hoc analysis. * p<0.05; ** p<0.01; *** p<0.001; **** p<0.0001.

<u>Abbreviations</u>: ns..non-significant (p≥0.05).

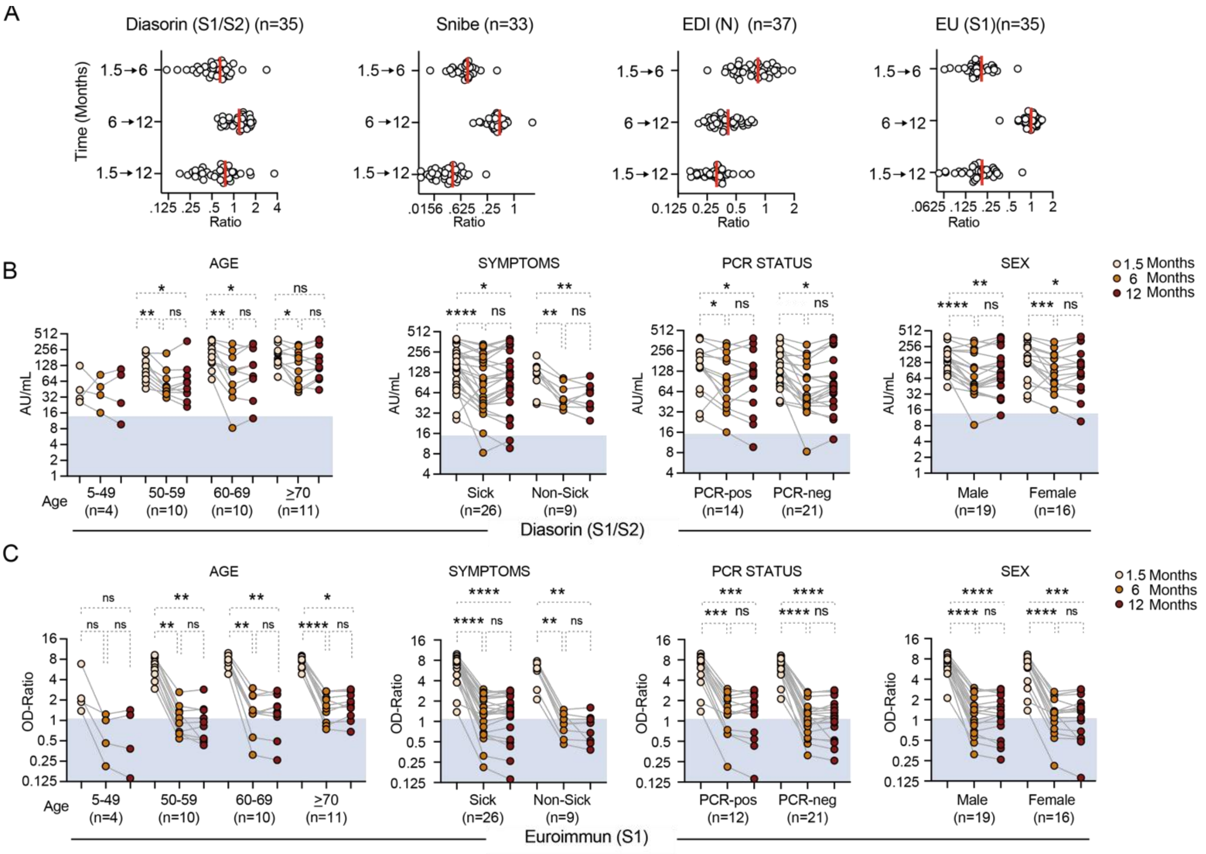

Supplementary Figure 2 (refers to *Figure 3*): **A**) Representative gating on spike peptide (S.Pep1 (N) restimulated PBMCs. **B**) Gating stragegy for CD137 and CD154 in CD3^{+}CD4^{+} cells in SARS-CoV-2 positive cells defined as previously PCR-positive or at least two positive antibody tests 1.5 months after SARS-CoV-2 outbreak and controls defined as no positive PCR results and less then two positive antibody tests after 1.5 and 12 months after after SARS-CoV-2 outbreak, respectively.

Abbreviations: DMSO.. Dimethylsulfoxide

A

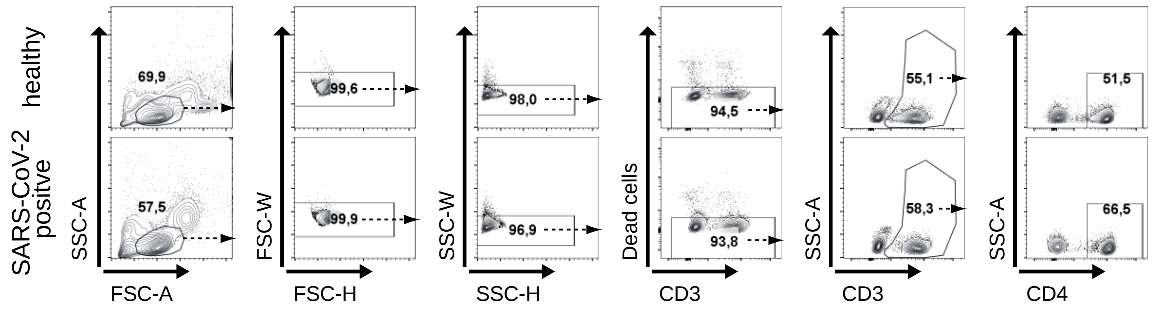

B

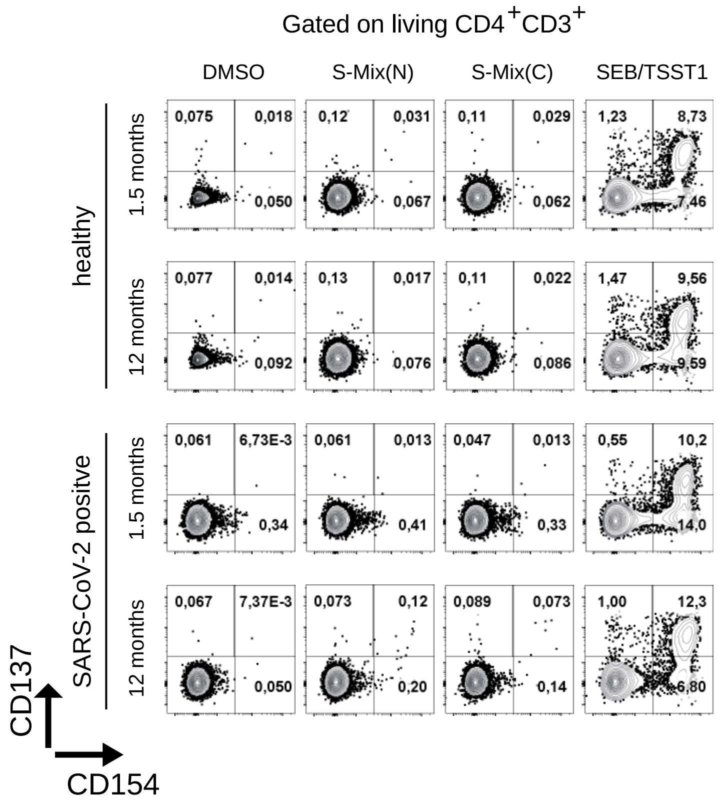

Supplementary Figure 3 (refers to *Figure 3*): SARS-CoV-2 specific T_H cell response of participants that were (**A-D**) seropositive but had no positive PCR test or (**E-F**) were tested positive either by PCR or by serum antibodies. **A**) % of 154+4-1BB+ T_H cells stimulated with DMSO or S.Pep.2 (C). **B**) Direct comparison of % of CD154+4-1BB+ T_H cells stimulated with or S.Pep.2 (C). **C, D**) % of 154+4-1BB+ T_H cells stimulated with **C**) S.Pep.1 (N) or **D**) S.Pep.2 (C) in SARS-CoV-2 negative (Ctrl) and positive (SARS-CoV-2+) participants. **E, F**) % of 154+4-1BB+ T_H cells in PCR-positive antibody negative vs. PCR-negative antibody-positive participants stimulated with **E**) S.Pep.1 (N) or **F**) S.Pep.2 (C) in SARS-CoV-2 negative (Ctrl) and positive (SARS-CoV-2+) participants. * $p<0.05$; ** $p<0.01$; *** $p<0.001$; **** $p<0.0001$.

Abbreviations: AB.. antibody; Ctrl.. control; DMSO.. Dimethylsulfoxide;

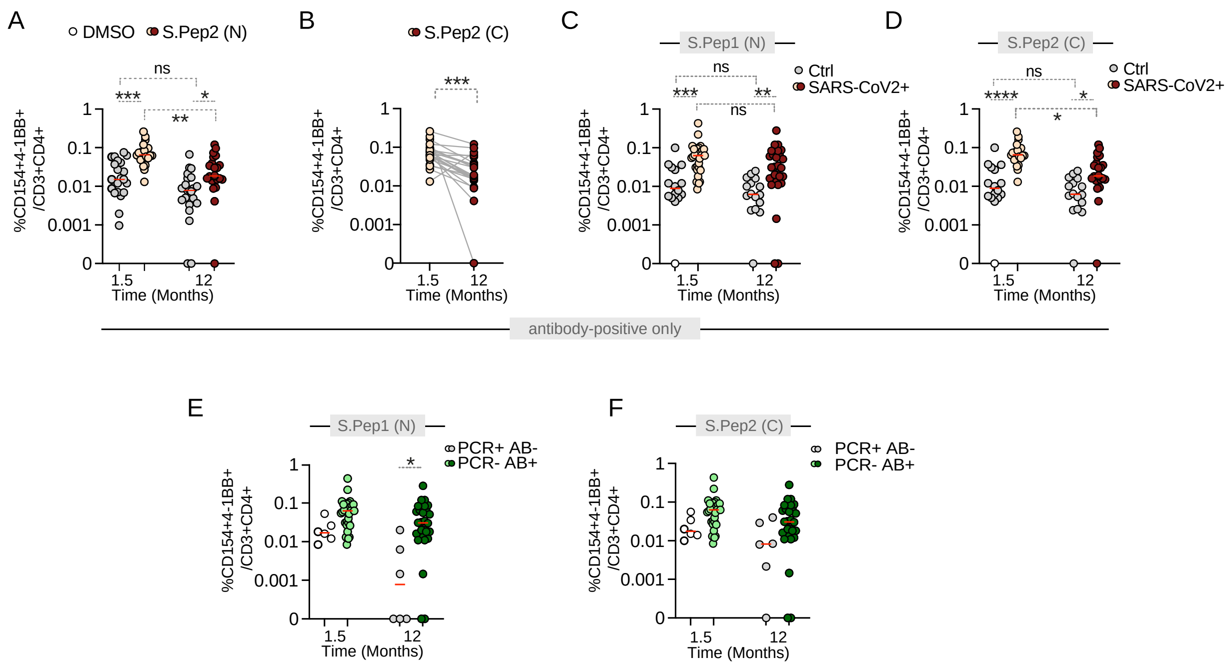

Supplementary Figure 4 (refers to *Figure 4*): Single populations of indicated markers.

Abbreviations: CD.. cluster of differentiation; IL.. interleukin; INFγ.. interferon; TNF.. tumor necrosis factor.

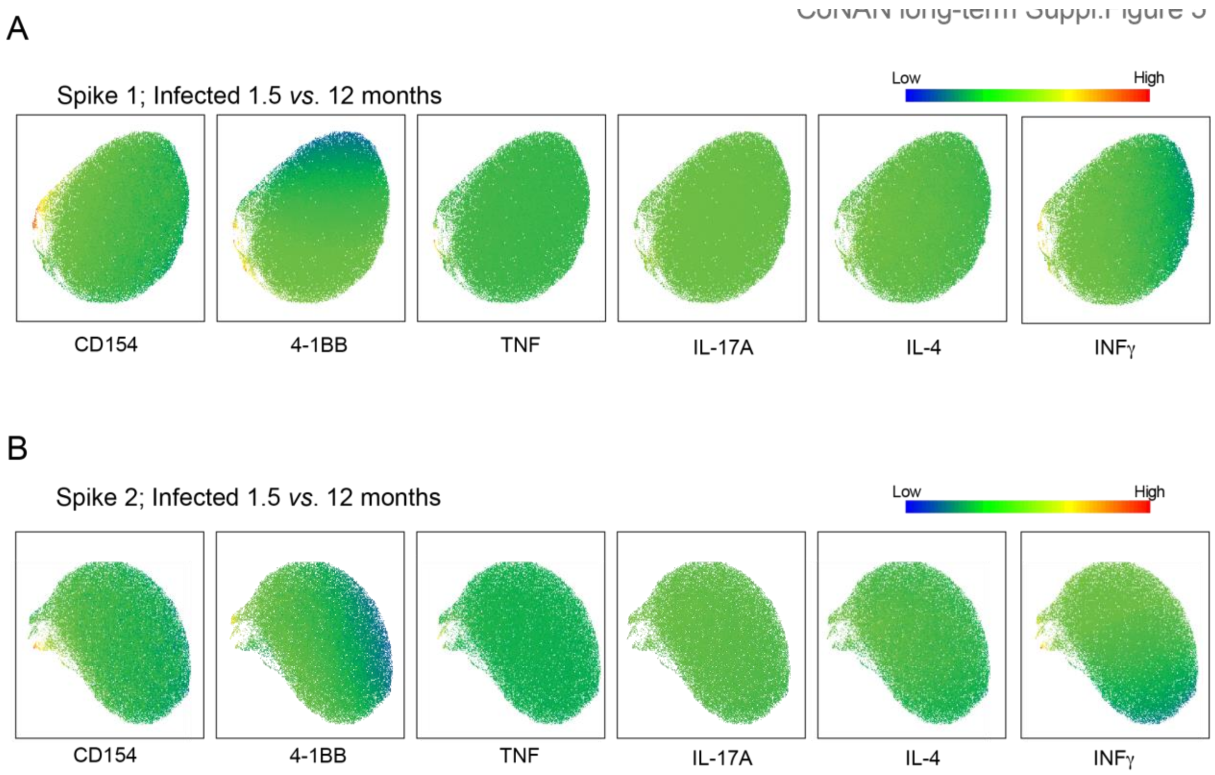

Supplementary Figure 5 (refers to *Figure 5*): Spearman Correlation analysis of SARS-CoV-2 specific T_H cells ($CD3^+CD4^+CD154^+4\text{-}1BB^+$) after S.Pep-1 (N) stimulation with all serological test results regardless of positive or negative antibody test results as indicated. Comparisons were the different combinations of the 1.5-month and 12-month time points after the SARS-CoV-2 outbreak in Neustadt am Rennsteig. Orange..1.5-month antibody data; Dark red.. 12-month antibody data.

Abbreviations: mo..month; TH.. T helper cells. Dots indicate individual participants.

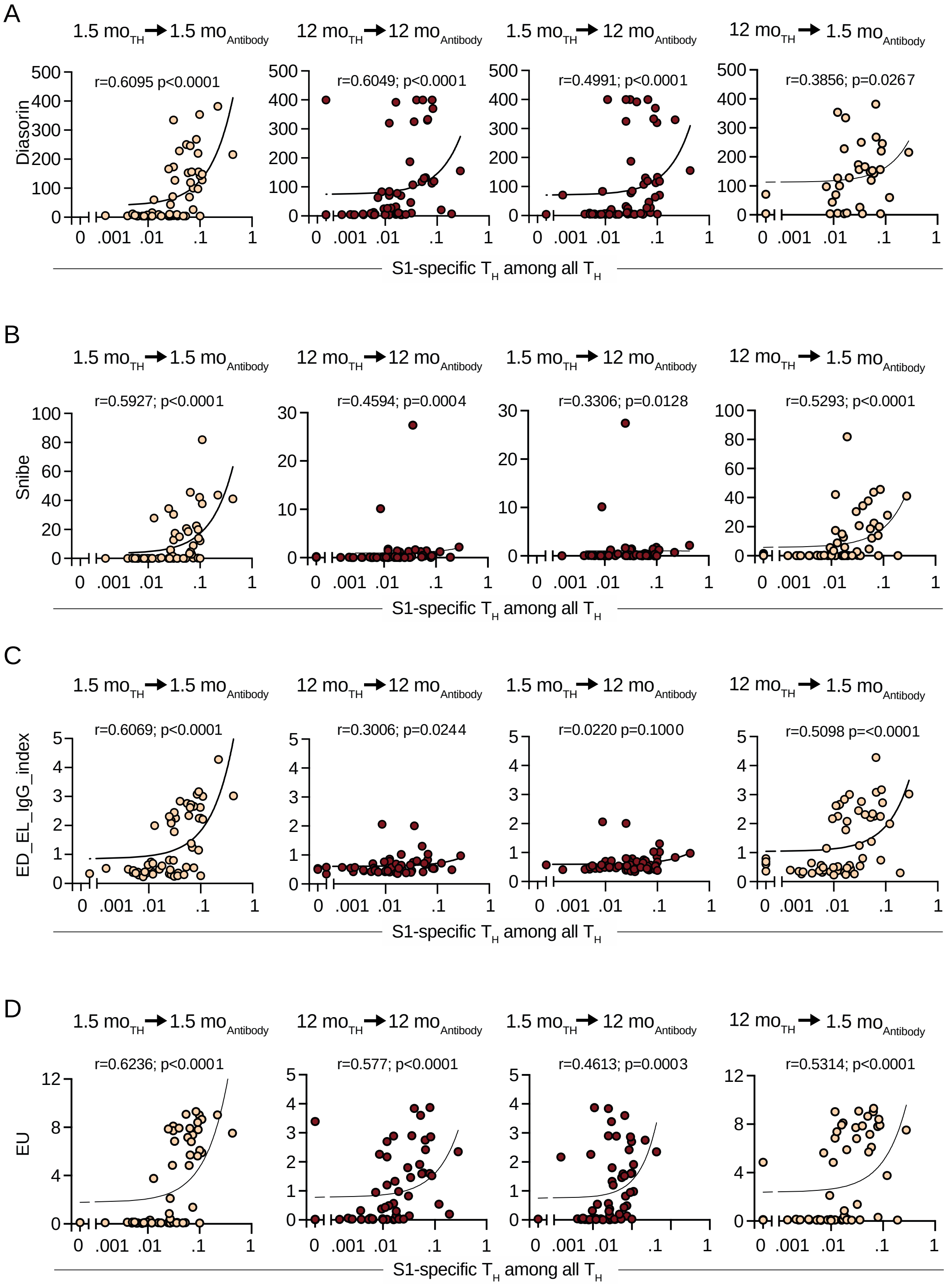

Supplementary Table 1: Sensitivities and specificities of the applied tests as provided by the manufacturer.

**Test and manufacturer**	**Instrument and manufacturer**	**Target**	**Sensitivity**	**Specificity**	**Note**
**Enzyme-linked immunosorbent assays**					
EDI Novel Coronavirus COVID-19 IgG ELISA *Epitope Diagnostics, San Diego, USA*	Immunomat *Virion\Serion, Würzburg, Germany*	Nucleocapsid	98·4% (184/187) 95% CI: 95·4%; 99·5%	99·8% (623/624) 95% CI: 99·1%; 99·97%	no information about time of positivity has been given
Euroimmun Anti-SARS-CoV-2-ELISA (IgG) *Euroimmun, Lübeck, Germany*	Tecan Sunrise *Tecan, Männedorf, Switzerland*	S1 domain	90·0% (27/30) 95% CI: 74·4%; 96·5%	100% (80/80) 95% CI: 95·4%; 100%	>21 days post-symptom onset
**Chemiluminescence assays**					
Liaison SARS-CoV-2 S1/S2 IgG CLIA *DiaSorin, Saluggia, Italy*	DiaSorin Liaison XL *DiaSorin, Saluggia, Italy*	S1/S2 domains	97·6% (40/41) 95% CI: 87·4%; 99.6%	99·3% (1082/1090) 95% CI: 98·6%; 99·6%	>14 days post-symptom onset
Maglumi 2019-nCoV IgG CLIA *Snibe, Shenzhen, China*	MAGLUMI 800 *Snibe, Shenzhen, China*	2019-nCoV recombinant antigen S+N	91·21% 95% CI: NA	100% 95% CI: NA	no information about time of positivity has been given
Elecsys Anti-SARS-CoV-2 ECLIA *Roche, Basel, Switzerland*	Cobas 8000 *Roche, Basel, Switzerland*	Nucleocapsid	100% (29/29) 95% CI: 88·3%; 100%	99·8% (5262/5272) 95% CI: 99·7%; 99·9%	≥14 days post-symptom onset

Supplementary Table 2: (refers to *Figure 5*): Spearman Correlation analysis of SARS-CoV-2 specific T_H cells (CD3+CD4+CD154+4-1BB+) after S.Pep-2 (C) stimulation with positive serological test only. Comparisons were the different combinations of the 1.5-month and 12-month time points after the SARS-CoV-2 outbreak in Neustadt am Rennsteig. red.. fair statistically significant correlation

Comparison	Spearman r [95%-Confidence Intervall]	p-value
**DiaSorin Liaison SARS-CoV-2 S1/S2 IgG CLIA**		
1.5 months (Antibody)→ 1.5 months (T_H)	0.27 [-0.14 to 0.60]	0.1770
12 months (Antibody)→ 12 months (T_H)	0.27 [-0.04 to 0.63]	0.1514
1.5 months (Antibody)→ 12 months (T_H)	0.34 [-0.04 to 0.63]	0.0677
12 months (Antibody)→ → 1.5 months (T_H)	-0.01 [-0.40 to 0.38]	0.9475
**Snibe Maglumi 2019-nCoV IgG**		
1.5 months (Antibody)→ 1.5 months (T_H)	0.25 [-0.18 to 0.59]	0.2384
12 months (Antibody)→ 12 months (T_H)	0.10 [-0.52 to 0.65]	0.7614
1.5 months (Antibody)→ 12 months (T_H)	-0.20 [-0.70 to 0.44]	0.5431
12 months (Antibody)→ → 1.5 months (T_H)	0.06 [-0.35 to 0.46]	0.7683
**EDI Novel Coronavirus COVID-19 IgG**		
1.5 months (Antibody)→ 1.5 months (T_H)	0.53 [0.16 to 0.77]	0.0064
12 months (Antibody)→ 12 months (T_H)	-0.87 [not available]	0.6667
1.5 months (Antibody)→ 12 months (T_H)	-1.00 [not available]	0.3333
12 months (Antibody)→ → 1.5 months (T_H)	-0.13 [-0.58 to 0.29]	0.5389
**Euroimmun Anti-SARS-CoV-2-ELISA IgG**		
1.5 months (Antibody)→ 1.5 months (T_H)	0.25 [-0.16 to 0.59]	0.2115
12 months (Antibody)→ 12 months (T_H)	0.21 [-0.24 to 0.59]	0.3442
1.5 months (Antibody)→ 12 months (T_H)	-0.02 [-0.45 to 0.41]	0.9225
12 months (Antibody)→ → 1.5 months (T_H)	-0.12 [-0.49 to 0.30]	0.5788
**Roche Elecsys Anti-SARS-CoV-2 ECLIA**		
1.5 months (Antibody)→ 1.5 months (T_H)	-0.06 [-0.42 to 0.33]	0.7722
12 months (Antibody)→ 12 months (T_H)	-0.12 [-0.47 to 0.27]	0.5466
1.5 months (Antibody)→ 12 months (T_H)	0.25 [-0.14 to 0.57]	0.1953
12 months (Antibody)→ → 1.5 months (T_H)	0.25 [-0.14 to 0.57]	0.1953

<u>Supplementary Table 3:</u> (refers to *Figure 5*): Spearman Correlation analysis of SARS-CoV-2 specific T_H cells after S.Pep-2 (C) stimulation with all serological test results regardless of positive or negative antibody test results as indicated. Comparisons were the different combinations of the 1.5-month and 12-month time points. blue.. moderately strong & statistically significant correlation, red.. fair & statistically significant correlation.

	**Spearman r [95%-Confidence Intervall]**	**p-value**
**DiaSorin Liaison SARS-CoV-2 S1/S2 IgG CLIA**		
1.5 months (Antibody)→ 1.5 months (T_H)	0.60 [0.40 to 0.75]	<0.0001
12 months (Antibody)→ 12 months (T_H)	0.44 [0.19 to 0.63]	0.0008
1.5 months (Antibody)→ 12 months (T_H)	0.55 [0.32 to 0.71]	<0.0001
12 months (Antibody)→ → 1.5 months (T_H)	0.16[-0.12 to 0.41]	0.2396
**Snibe Maglumi 2019-nCoV IgG**		
1.5 months (Antibody)→ 1.5 months (T_H)	0.62 [0.42 to 0.76]	<0.0001
12 months (Antibody)→ 12 months (T_H)	0.19 [0.09 to 0.44]	0.1698
1.5 months (Antibody)→ 12 months (T_H)	0.39 [0.14 to 0.60]	0.0028
12 months (Antibody)→ → 1.5 months (T_H)	0.22 [-0.05 to 0.46]	0.1019
**EDI Novel Coronavirus COVID-19 IgG**		
1.5 months (Antibody)→ 1.5 months (T_H)	0.64 [0.45 to 0.78]	<0.0001
12 months (Antibody)→ 12 months (T_H)	0.02 [-0.26 to 0.28]	0.9130
1.5 months (Antibody)→ 12 months (T_H)	0.25 [-0.02 to 0.49]	0.0651
12 months (Antibody)→ → 1.5 months (T_H)	0.26 [-0.01 to 0.50]	0.0503
**Euroimmun Anti-SARS-CoV-2-ELISA IgG**		
1.5 months (Antibody)→ 1.5 months (T_H)	0.62 [0.43 to 0.77]	<0.0001
12 months (Antibody)→ 12 months (T_H)	0.42 [0.17 to 0.62]	0.0014
1.5 months (Antibody)→ 12 months (T_H)	0.47 [0.23 to 0.66]	0.0003
12 months (Antibody)→ → 1.5 months (T_H)	0.27 [0.002 to 0.51]	0.0421
**Roche Elecsys Anti-SARS-CoV-2 ECLIA**		
1.5 months (Antibody)→ 1.5 months (T_H)	0.61 [0.41 to 0.76]	<0.0001
12 months (Antibody)→ 12 months (T_H)	0.20 [-0.08 to 0.45]	0.1434
1.5 months (Antibody)→ 12 months (T_H)	0.59 [0.38 to 0.74]	<0.0001
12 months (Antibody)→ → 1.5 months (T_H)	0.24 [-0.04 to 0.48]	0.0809
